# Health related quality of life after gastric bypass or intensive lifestyle intervention: a controlled clinical study

**DOI:** 10.1186/1477-7525-11-17

**Published:** 2013-02-13

**Authors:** Tor Ivar Karlsen, Randi Størdal Lund, Jo Røislien, Serena Tonstad, Gerd Karin Natvig, Rune Sandbu, Jøran Hjelmesæth

**Affiliations:** 1 Morbid Obesity Centre, Vestfold Hospital Trust, Tønsberg, Norway; 2 Department of Health and Nursing Sciences, University of Agder, Grimstad, Norway; 3 Department of Biostatistics, Institute of Basic Medical Sciences, University of Oslo, Oslo, Norway; 4 School of Public Health, Loma Linda University, Loma Linda, CA, USA; 5 Department of Public Health and Primary Health Care, University of Bergen, Bergen, Norway

**Keywords:** Quality of life, Bariatric surgery, Lifestyle modification

## Abstract

**Background:**

There is little robust evidence relating to changes in health related quality of life (HRQL) in morbidly obese patients following a multidisciplinary non-surgical weight loss program or laparoscopic Roux-en-Y Gastric Bypass (RYGB). The aim of the present study was to describe and compare changes in five dimensions of HRQL in morbidly obese subjects. In addition, we wanted to assess the clinical relevance of the changes in HRQL between and within these two groups after one year. We hypothesized that RYGB would be associated with larger improvements in HRQL than a part residential intensive lifestyle-intervention program (ILI) with morbidly obese subjects.

**Methods:**

A total of 139 morbidly obese patients chose treatment with RYGB (n=76) or ILI (n=63). The ILI comprised four stays (seven weeks) at a specialized rehabilitation center over one year. The daily schedule was divided between physical activity, psychosocially-oriented interventions, and motivational approaches. No special diet or weight-loss drugs were prescribed. The participants completed three HRQL-questionnaires before treatment and 1 year thereafter. Both linear regression and ANCOVA were used to analyze differences between weight loss and treatment for five dimensions of HRQL (physical, mental, emotional, symptoms and symptom distress) controlling for baseline HRQL, age, age of onset of obesity, BMI, and physical activity. Clinical relevance was assessed by effect size (ES) where ES<.49 was considered small, between .50-.79 as moderate, and ES>.80 as large.

**Results:**

The adjusted between group mean difference (95% CI) was 8.6 (4.6,12.6) points (ES=.83) for the *physical dimension*, 5.4 (1.5–9.3) points (ES=.50) for the *mental dimension,* 25.2 (15.0–35.4) points (ES=1.06) for the *emotional dimension,* 8.7 (1.8–15.4) points (ES=.37) for the measured *symptom distress,* and 2.5 for (.6,4.5) fewer *symptoms (ES=.56)*, all in favor of RYGB. Within-group changes in HRQOL in the RYGB group were large for all dimensions of HRQL. Within the ILI group, changes in the emotional dimension, symptom reduction and symptom distress were moderate. Linear regression analyses of weight loss on HRQL change showed a standardized beta-coefficient of –.430 (p<.001) on the physical dimension, –.288 (p=.004) on the mental dimension, –.432 (p<.001) on the emotional dimension, .287 (p=.008) on number of symptoms, and .274 (p=.009) on reduction of symptom pressure.

**Conclusions:**

Morbidly obese participants undergoing RYGB and ILI had improved HRQL after 1 year. The weaker response of ILI on HRQL, compared to RYGB, may be explained by the difference in weight loss following the two treatments.

**Trial registration:**

Clinical Trials.gov number NCT00273104

## Introduction

Morbid obesity is understood as a body mass index (BMI) ≥40 kg/m^2^ or BMI ≥35 kg/m^2^ with comorbidities [1]. Roux-en-Y Gastric Bypass (RYGB) is an effective and commonly used [2] surgical procedure for treatment of morbid obesity. Although the majority of patients may prefer non-surgical intervention, bariatric surgery has been shown to be more effective than lifestyle intervention at improving weight loss and obesity associated morbidities [3,4].

Improving patients’ health-related quality of life (HRQL) is an important treatment goal. This concept refers to how well an individual functions in daily life and their perceived well-being [5]. In accordance with the World Health Organization’s multidimensional definition of health [6], we conceptualize HRQL as encompassing physical, mental and emotional dimensions as well as the burden of obesity specific symptoms.

Few studies have addressed the comparative effects of bariatric surgery and lifestyle intervention on HRQL. The Swedish Obese Subjects research program (SOS), a 10-year non-randomized controlled longitudinal study, compared patients undergoing various bariatric procedures (n=655) with patients (n=621) undergoing conventional weight-loss treatment [7]. Notably, treatment for the conventionally treated patients was not standardized and treatment regimens varied according to local practices. The Swedish study showed that patients who chose surgery lost about 15 times more weight than non-surgically treated patients, mean (SD) loss of 19.7 (15.8) kg vs. 1.3 (13.8) kg. In addition, the study reported that the surgical groups sustained positive outcomes in HRQL compared to non-surgical matched controls. This effect was mainly explained by weight loss. A two-year controlled non-randomized study by Kolotkin et al. [8] found significant improvements in HRQL in patients undergoing RYGB (n=308) compared to a control group of patients who sought but did not undergo RYGB (n=253) and a population-based group of obese individuals (n=272).

Notably, neither study predefined the lifestyle intervention for the non-surgical groups making comparison between bariatric surgery and comprehensive lifestyle programs difficult. The evidence thus remains limited regarding HRQL following RYGB in comparison to specific comprehensive and multidisciplinary lifestyle interventions. In addition, most studies of HRQL in morbid obesity have focused on the physical and mental aspects, applying generic instruments of HRQL measurement. However, the development of obesity-specific HRQL instruments enables additional analyses of the emotional and symptomatic dimensions. Furthermore, only a few studies of HRQL in the morbidly obese have calculated the effect size (ES) of change in HRQL, which underscores the clinical relevance of the various treatments.

An earlier report [9] demonstrated that type 2 diabetes and obesity-related cardiovascular risk factors such as hypertension and hyperlipidemia were improved after both RYGB and a pre-defined part residential multidisciplinary non-surgical intensive lifestyle-intervention program (ILI). However, the improvements were greatest in those patients treated with RYGB. This study did not evaluate the individuals’ subjective notion of well-being or how their daily life functioned following these two interventions.

The aim of the present study was to describe and compare changes in five dimensions of HRQL (physical, mental, emotional, number of symptoms, and symptom distress) following RYGB and ILI in morbidly obese subjects. Secondarily, we wanted to assess the clinical relevance of the changes in HRQL between and within these two groups after one year. We hypothesized that RYGB would be associated with larger improvements of HRQL than ILI in morbidly obese subjects.

## Methods and procedures

This is a preplanned analysis of data from the MOBIL-study (Morbid Obesity treatment, Bariatric surgery versus Intensive Lifestyle intervention, Clinical Trials.gov number NCT00273104), a non-randomized controlled study designed to compare the effects of bariatric surgery and intensive lifestyle intervention on various comorbidities, eating behavior and HRQL.

**Figure 1:**
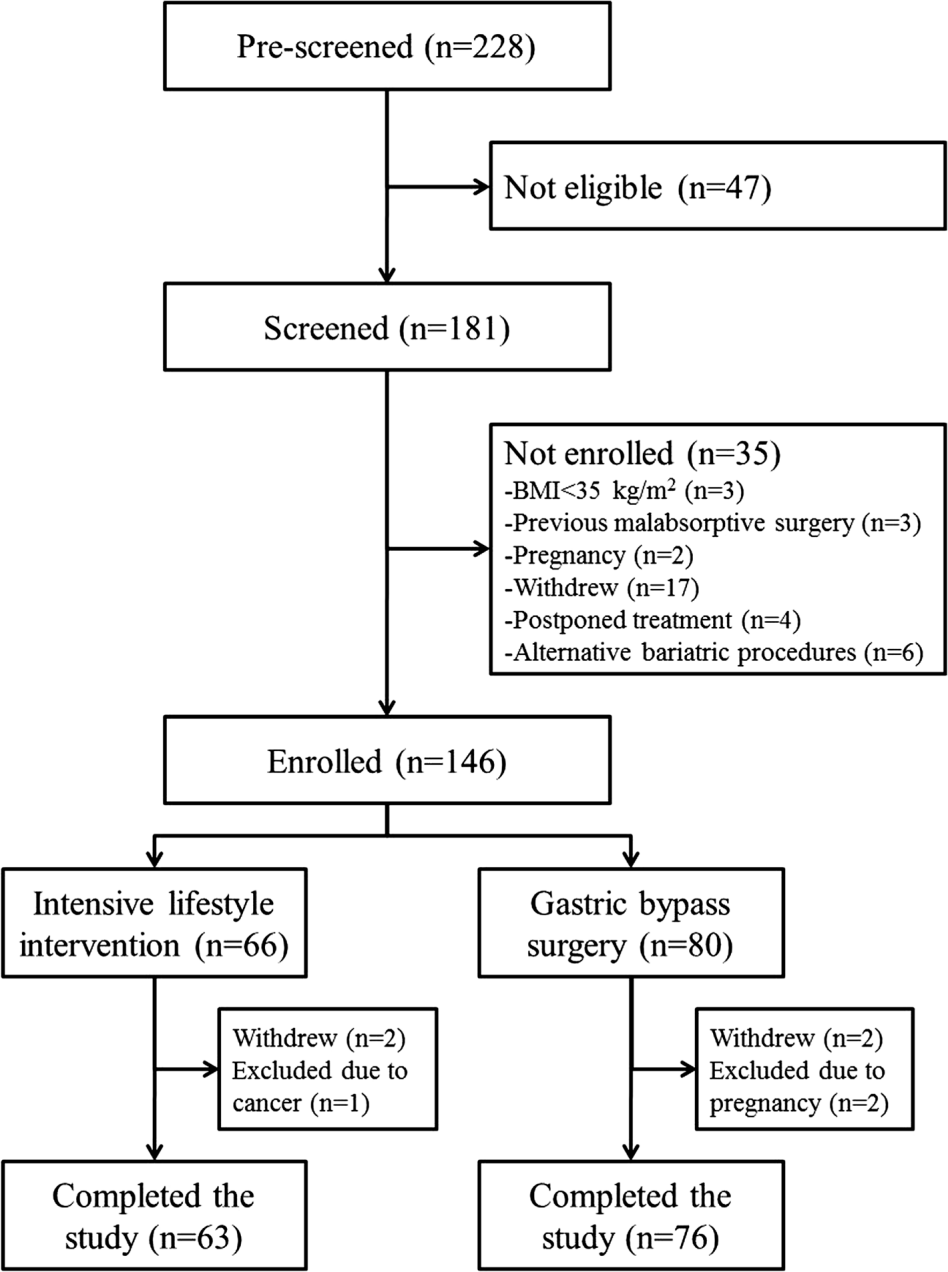
Flow of patients.

A total of 228 patients were screened, with 47 found not to be eligible. Of the remaining 181 participants 35 were not enrolled, leaving 146 in the study (Figure 1, flow of participants).

During the screening procedure all eligible patients underwent a thorough assessment at the Morbid Obesity Center by a multidisciplinary team consisting of an internist, a dietician, a physiotherapist and a trained “obesity” nurse. Patients were provided information about the possible risks and benefits of an operation and also encouraged to incorporate their own values and preferences into the decision-making process. If no contraindication against surgery existed, the patient and the physician together agreed upon the most appropriate choice of therapy; either surgical or conservative [10].

A previous report [9] showed a mean (SD) 1-year weight loss of 30 (8)% of initial body weight in the RYGB group and 8 (9)% in the ILI group. This corresponds to a mean (SD) loss of excess weight above 25 kg/m^2^ of 67% (18) and 20% (23) (P<0.001) respectively. The patients in the RYGB group lost a mean (SD) of 14.0 (4.1) BMI points and the ILI group 3.7 (4.2) BMI points. The number of subjects in the RYGB group and ILI group who either moved from being inactive to active (12 vs 18), stayed inactive or active (57 vs 32), or moved from being active to inactive (4 vs 5), differed significantly between the groups. Overall, there was a greater increase in the physical activity level of the lifestyle group compared to the surgery group.

The Norwegian Regional Ethics Committee for Medical Research approved the study protocol (S-05175), and the study was performed in accordance with the Declaration of Helsinki. All participants gave informed written consent before enrolment.

### Variables, measurement and outcomes

The main outcome in the current analysis was the change in each of the five dimensions of HRQL, conceptualized as encompassing physical, mental and emotional dimensions, as well as the number and burden of obesity-specific symptoms. Since we aimed to compare the effect of two treatment methods, and since entering both weight loss and type of treatment into the same statistical analysis led to multicollinearity (r=.81), weight loss was excluded from the multiple regression analyses of covariance (ANCOVA). Variables that were considered possible confounders included age and BMI prior to intervention, age at the onset of obesity, physical activity and HRQL-score prior to intervention. Three questionnaires were used to measure HRQL: the Medical Outcome Study 36 – Item Short Form Health Survey (SF-36), the Obesity and Weight-Loss Quality of Life (OWLQOL), and the Weight Related Symptom Measure (WRSM).

#### Medical Outcome Study 36 – Item Short Form Health Survey (SF-36)

SF-36 is a commonly used generic measure of HRQL based on 36 questions or items [11-13]. Item 2 is not included in the scoring of the instrument [13]. The remaining 35 items form eight subscales (physical function, role physical, bodily pain, general health, role emotional, social function, vitality, and mental health) which can be combined into two summary scores; the physical and mental dimensions [14]. As the validity of the subscales in morbidly obese patients is uncertain [15,16] we studied the physical and mental dimensions. The calculations were performed as recommended by the scale authors [14], using Norwegian norms [17] and oblique factor scores to account for the correlation between the two HRQL-dimensions. The scores were calculated by multiplying each subject’s SF-36 subscale z score by its respective factor coefficient and then standardizing each to a T score with a mean of 50 and a standard deviation of 10 [14]. Both scales were set to a range from 0–100, where higher scores indicate better HRQL.

#### Obesity and Weight-Loss Quality of Life (OWLQOL)

The OWLQOL [18,19] primarily measures emotions and feelings [20,21] which are believed to result from being obese and trying to lose weight. The instrument consists of 17 statements about weight-related feelings and emotions which are rated on a seven-point scale that ranges from 0 (“not at all”) to 6 (“a very great deal”). The 17 items of the OWLQOL form a scale ranging from 0–102, with higher scores indicating greater emotional HRQL.

#### Weight Related Symptom Measure (WRSM)

The WRSM [18,19] measures 20 obesity-specific symptoms using two different sets of items. The first set assesses whether or not the patient is experiencing specific symptoms. The scoring of this set of items creates an additive scale summing up the number of symptoms, ranging from 0–20. The second set of items concerns the distress of the symptoms, with values from 0 (“not at all”) to 6 (“bothers a very great deal”). They form a symptom distress scale ranging from 0–120, where higher scores indicate greater symptom distress. Both the OWLQOL and the WRSM were obtained with permission from the Seattle Quality of Life Group, University of Washington.

In sum, the three HRQL questionnaires constitute five different measurements of HRQL; physical dimension (SF-36), mental dimension (SF-36), emotional dimension (OWLQOL), number of obesity symptoms (WRSM), and distress of obesity symptoms (WRSM).

Changes in scores between two time-points or groups can be statistically significant. An important follow-up question is whether the changes are clinically relevant. There are different approaches to addressing this. Here we have chosen the effect size (ES) to grade the efficiency of surgical versus nonsurgical treatment [22,23].

Physical activity was assessed through structured interviews performed by registered dieticians. Time spent performing light (e.g. casual walking), moderate (e.g. brisk walking) and vigorous (e.g. jogging) intensity aerobic physical activities for periods of 10 minutes or more was recorded. Participants who performed 150 minutes or more per week of moderately intense aerobic physical activities were considered to be physically active, as were those participants who performed 60 minutes or more per week of vigorously intense aerobic physical activities [24].

### Participants

A total of 139 patients completed the MOBIL-study (Figure 1). At baseline, all patients in both the RYGB group (n=76) and the ILI group (n=63) completed the three HRQL instruments. At 1 year follow up 62 (82%) participants in the RYGB group and 48 (76%) in the ILI group had completed the questionnaires. In order to assess the representativeness of the sample at the end of the study we used an independent samples *t*-test to compare differences between patients not completing the questionnaires at the end of the study versus completers. Patients who did not complete the questionnaires after 1 year (n=29) were comparable with those who did (n=110) with regards to baseline HRQL, gender, age, body weight, employment status, and weight loss after 1 year (data not shown).

### Interventions

During follow-up, patients allocated to RYGB were examined by a bariatric surgeon 6 weeks after surgery, while patients were seen by a dietician quarterly, usually in groups of 12–16. The patients in the ILI group were admitted to a rehabilitation center specializing in the care of morbidly obese patients. The aim was to attain a sustained 1-year weight loss ≥10%. Each patient was encouraged to increase their physical activity and to normalize eating habits. The program intended to increase each patient’s self-efficacy in dealing with their weight problem, as well as an improvement in self-esteem.

**Figure 2:**
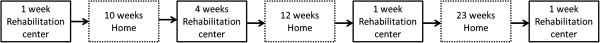
Schedule of stays during the 1-year intensive lifestyle intervention program at the rehabilitation centre.

The 1-year lifestyle program comprised four stays at the rehabilitation center – three 5-day stays in weeks 1, 26, and 51, and a four-week stay from weeks 13–17 (Figure 2). The daily schedule was divided between organized daily physical activity (3–4 hours) and various psychosocially-oriented interventions combined with a motivational approach both in group sessions and individual sessions (3–4 hours). These sessions were supervised by a medical doctor, nutritionists, physiotherapists and mental health-trained nurses. No special diet or weight-loss drugs were prescribed, but patients were encouraged to follow the guidelines of the Norwegian National Council of Nutrition [25], which recommends that the daily intake of protein, fat, carbohydrate and alcohol should account respectively for 10–20, <30, 50–60, and <5% of energy consumed. In addition, the patients were asked to reduce their daily total energy intake, but not using calorie-counting. Outside of these stays patients were contacted by phone once every 2 weeks. They were also encouraged to self-monitor their eating habits and physical activities in a pre-fabricated diary, as well as to consult their general practitioner for weight measurement and follow-up every four weeks.

### Statistical methods

Data are presented as mean (SD) or n (%) unless otherwise stated. Skewed data were transformed to approximate normality using natural logarithms. To assess the reliability of the HRQL-scales we calculated Cronbach’s alpha coefficients.

After applying Little’s test of randomness of missing data, missing values (SF-36:23.5%, OWLQOL:24.5%, WRSM:23.7%) were imputed using multiple imputation. The imputation model consisted of the HRQL-scores, physical activity at baseline and 1 year, and age of onset of obesity as predictor and imputation variables, and treatment, gender, age, baseline BMI, marital status, employment, and education as predictor variables. Through a fully conditional specification model, applying linear regression as the prediction method for scale variables and two-way interactions for categorical variables, we generated twenty complete datasets for each of the HRQL-scores with 10 iterations per dataset. The statistical analyses were performed on each complete dataset, and thereafter the multiple analyses results were combined to achieve single estimates. The combined estimates are presented. Observing the fraction of missing information, relative increase variance, and relative efficiency, the imputed data-sets (n=139) were comparable with the original data-set (n=110) in terms of the imputed variables (data not shown).

Within-group analyses in both groups were performed using paired samples *t*-test. Between-group comparisons at baseline were analyzed using independent samples *t*-test for continuous variables and *χ*^2^ for categorical variables.

Within groups ES was calculated as the mean HRQL change score between 1 year and baseline divided by the standard deviation of the baseline HRQL. Between groups ES was calculated as the difference in mean HRQL change score between groups at 1 year divided by the standard deviation of baseline HRQL [22,23]. An ES from .20–.49 was considered small, .50–.79 as moderate, and greater than .80 as large [22,23].

In order to avoid problems of regression towards the mean [26,27], we applied one-way between-group analyses of covariance (ANCOVA) to compare the effect of RYGB and lifestyle intervention on five dimensions of HRQL. Age at baseline, age at the onset of obesity, BMI at baseline, physical activity at baseline, and baseline HRQL-scores were used as covariates in each of the five analyses [28]. Assessments of normality, linearity, homogeneity of variance and regression slopes were conducted to ensure assumptions for the ANCOVA. The unadjusted changes from baseline in the RYGB group and ILI group, together with the adjusted between group differences (95% CI), are reported. To account for the percentage explained variance in the dependents, calculations of partial eta squared (ηp^2^) were performed. To test the effect of weight reduction (instead of treatment choice) on HRQL, multiple linear regression analyses were conducted with each of the 12 months HRQL changes (physical, mental, emotional, number of obesity symptoms, and symptom distress) as dependents, with gender, age at baseline, age at the onset of obesity, BMI at baseline, physical activity at baseline, and weight change in per cent of baseline weight as independents. Throughout, we report two-tailed P values, with P<.05 was considered to be statistically significant. The statistical analysis was conducted using SPSS v.18.0.

## Results

### Internal consistency

The inter-item analyses showed Cronbach’s alpha coefficients >.80, indicating that intercorrelations among the items is high and that there is a high reliability for all of the HRQL-scales (physical, mental, and emotional dimensions, number of symptoms, and symptom distress).

### Patients

Baseline demographic characteristics are summarized in Table 1. Compared to the ILI group, the patients in the RYGB group had a higher BMI (ES=.49), were younger (ES=.36), had earlier onset of obesity (ES=.47), and had lower physical (ES=.50) and emotional HRQL (ES=.42).

**Table 1:** Demographic, socioeconomic and clinical characteristics of 139 morbidly obese individuals who chose a part residential intensive lifestyle intervention program (ILI) or gastric bypass surgery (RYGB)

**Variable**	**Total**	**RYGB**	**ILI**	**P-value**
	**(n=139)**	**(n=76)**	**(n=63)**	
Women (n, %)	97 (70%)	53 (70%)	44 (70%)	.569
Age (years), mean (SD)	46 (11)	43 (11)	47 (11)	.021
Onset of obesity (n, %)				
<12 years	35 (25%)	25 (33%)	10 (16%)	
12-20 years	28 (20%)	17 (22%)	11 (17%)	
>20 years	76 (55%)	34 (45%)	42 (67%)	.003
BMI (kg/m^2^), mean (SD)	44 (6)	46 (6)	43 (5)	<.001
Married/cohabitant, (n, %)	83 (60%)	45 (60%)	38 (60%)	.895
Employment (n, %)	82 (59%)	40 (53%)	42 (67%)	.094
Length of education (n, %)				
Basic (<9 year)	32 (23%)	18 (24%)	14 (22%)	
Intermediate (9–12 year)	75 (54%)	44 (58%)	31 (49%)	
Higher (>12 year)	2 (23%)	14 (18%)	18 (29%)	.358
Physical activity (n, %)				
Low	115 (83%)	67 (88%)	48 (76%)	
High	24 (17%)	9 (12%)	15 (24%)	.063
Quality of life scores, mean (SD)				
Physical dimension ^a^	36 (10)	34 (10)	39 (10)	.018
Mental dimension ^a^	41 (11)	41 (11)	42 (11)	.690
Emotional dimension ^b^	36 (24)	32 (23)	42 (24)	.047
Number of symptoms ^c^	11 (4)	12 (4)	11 (4)	.343
Symptom distress ^d^	41 (21)	43 (21)	38 (20)	.173

(a) SF-36 (scale 0–100). (b) OWLQOL (scale 0–102). (c) WRSM (scale 0–20). (d) WRSM (scale 0–120).

#### Changes in the five main dimensions of HRQOL

Adjusted between group analyses, controlling for the effects of treatment, age at baseline, age at the onset of obesity, BMI at baseline, physical activity at baseline, and baseline HRQL-scores, showed that compared to the ILI group, the RYGB group had statistically significant higher adjusted mean improvement in all HRQL-measurements, especially the emotional dimension (Table 2). Based on calculations of ηp^2^, type of treatment predicted 19.7% of the variance (ES=.83) in the physical dimension change score, 9.8% (ES=.50) in the mental dimension change score, 22.6% (ES=1.06) in the emotional dimension change score, 7.7% (ES=.56) in the number of symptoms, and 8.1% (ES=.37) in the symptom distress change score.

**Table 2:** One way between-groups analysis of variance on five dimensions of HRQL in morbidly obese patients undergoing either RYGB or ILI

	**Changes from baseline**		**Adjusted between group difference, mean (95% CI)**	**P**	**ES**
	**RYGB**	**ILI**			
	**(n=76)**	**(n=63)**			
Physical dimension^a^	16.8 (9.7)	4.9 (9.4)	8.6 (4.6,12.6)	<.001	.83
Mental dimension^a^	9.6 (9.1)	3.5 (8.9)	5.4 (1.5,9.3)	.007	.50
Emotional dimension^b^	42.7 (25.5)	15.7 (21.7)	25.2 (15.0,35.4)	<.001	1.06
Number of obesity symptoms^c^	−5.3 (4.6)	−2.9 (4.7)	−2.3 (−4.5,-.6)	.012	.56
Symptom distress^d^	−25.2 (20.7)	−14.3 (16.5)	−8.7 (−15.4,-1.8)	.013	.37

Adjustments were made for age at the onset of obesity and baseline values of age, BMI physical activity level, and HRQL. (a) SF-36 (scale 0–100). (b) OWLQOL (scale 0–102). (c) WRSM (scale 0–20). (d) WRSM (scale 0–120). Statistical significance (P) and effect size (ES) are reported.

**Figure 3:**
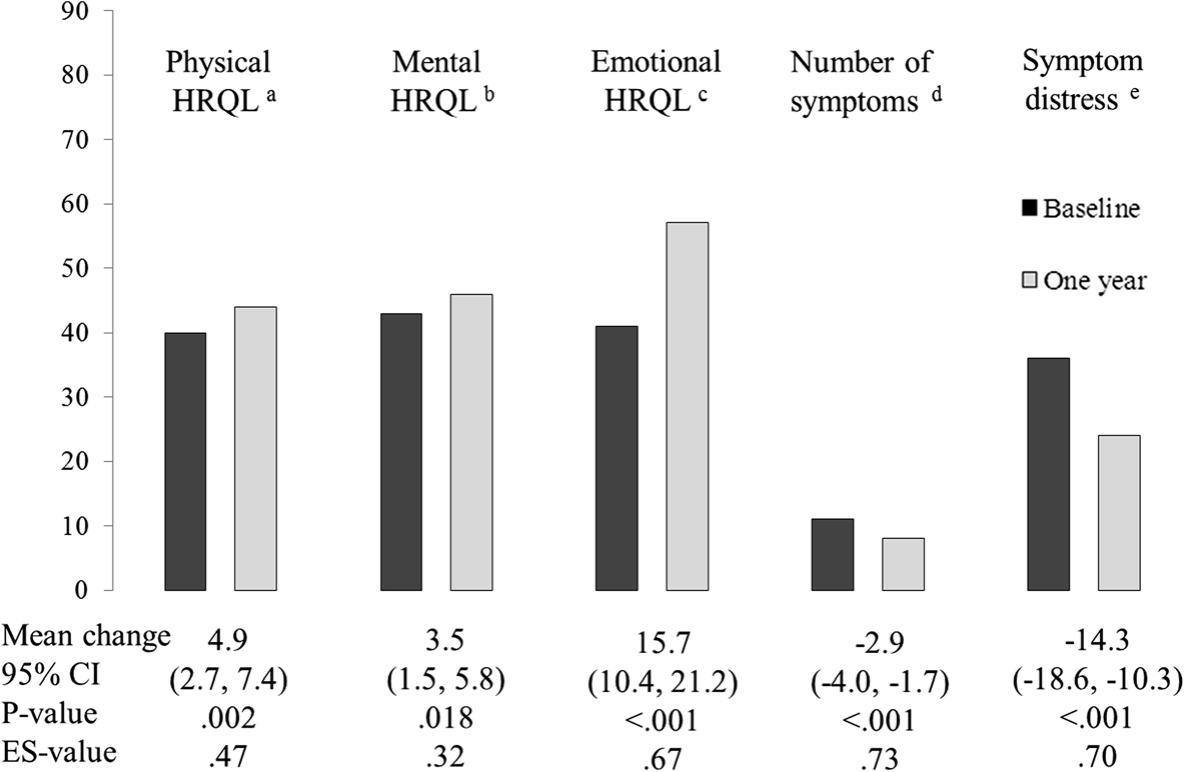
**Mean scores on five HRQL-scales at baseline and 1 year in morbidly obese patients who underwent a part residential intensive lifestyle intervention program (n=63).** (**a**) SF-36 Physical dimension (0–100). (**b**) SF-36 Mental dimension (0–100). (**c**) OWLQOL Emotional dimension (0–102). (**d**) WRSM symptom number score (0–20). (**e**) WRSM symptom severity score (0–120). Unadjusted mean change scores and 95% CI. P and ES-values for within-group changes.

**Figure 4:**
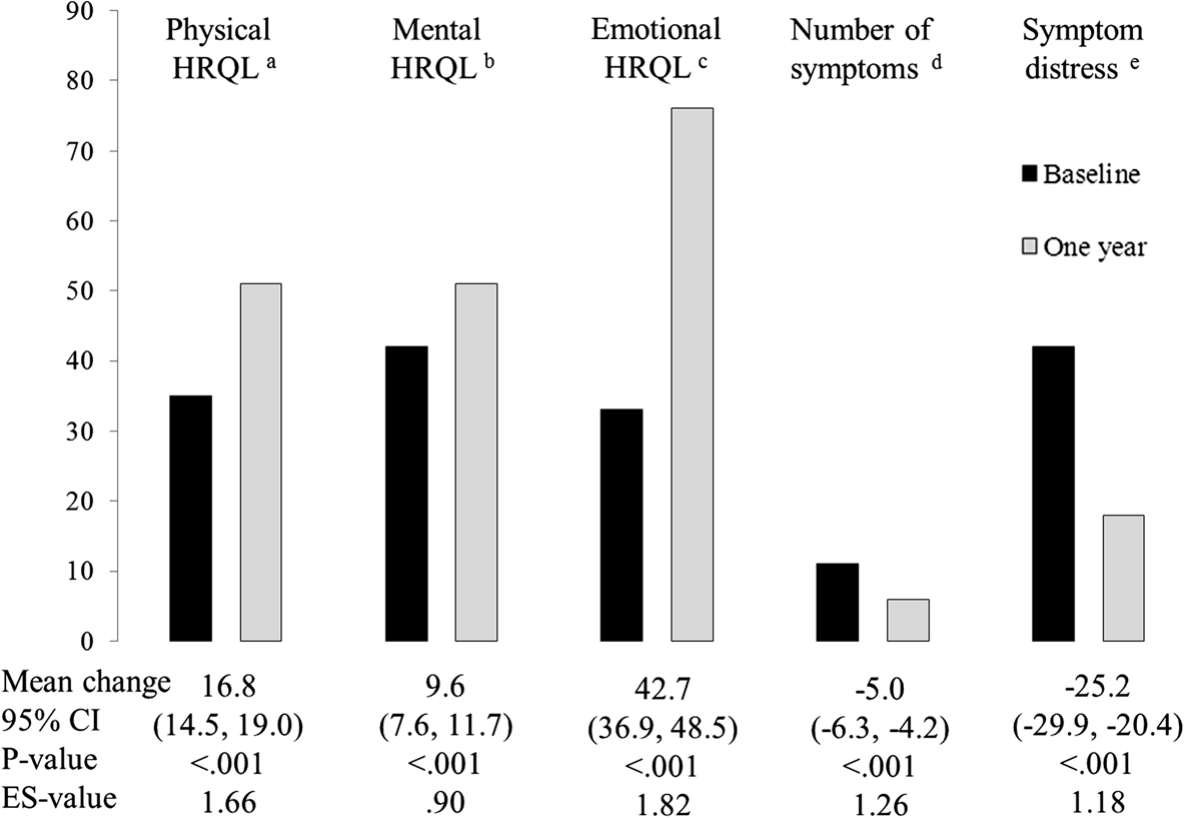
**Mean scores on five HRQL-scales at baseline and 1 year in morbidly obese patients who underwent RYGB (n=76). **(**a**) SF-36 Physical dimension (0–100). (**b**) SF-36 Mental dimension (0–100). (**c**) OWLQOL Emotional dimension (0–102). (**d**) WRSM symptom number score (0–20). (**e**) WRSM symptom distress score (0–120). Unadjusted mean change scores and 95% CI. P and ES-values for within-group changes.

Unadjusted within-group analyses showed that both groups reported improvements in all five HRQL-measurements (Figures 3 and 4). All effect sizes were large within the RYGB group and small to moderate within the ILI group.

#### Changes in self-reported symptom distress

Twenty common obesity specific health problems associated with obesity are listed in Table 3. Compared to the ILI group, the RYGB group showed greater improvement in ailments such as reduced physical stamina, joint pain, snoring, sleep problems, skin irritation, water retention, and foot problems. Only the improvements of physical stamina and joint pain showed large effect sizes between groups. On the other hand, the RYGB group reported higher sensitivity to cold (Table 3), and this difference was considered large.

**Table 3:** 1-year changes in reported symptom distress between groups of morbidly obese patients undergoing RYGB (n=76) or intensive lifestyle intervention (n=63)

**Symptom**	**RYGB**	**ILI**	**P**	**ES**
Physical stamina	−2.7 (2.5)	-.7 (2.6)	<.001	.913
Pain in the joints	−2.4 (2.2)	-.7 (2.0)	.002	.891
Snoring	−2.3 (2.2)	-. 8 (2.0)	.002	.721
Sensitivity to cold	1.4 (2.2)	.1 (1.9)	.005	.921
Skin irritation	−1.2 (1.9)	-.3 (1.8)	.032	.493
Sleep problems	−1.3 (2.2)	-.4 (2.2)	.043	.406
Water retention	−1.5 (1.9)	-.6 (2.0)	.047	.456
Foot problems	−2.2 (2.6)	−1.1 (2.5)	.050	.541
Back pain	−1.6 (2.0)	-.9 (1.7)	.071	.350
Tiredness	−1.6 (2.2)	-.7 (2.2)	.089	.460
Shortness of breath	−2.2 (2.1)	−1.4 (1.9)	.093	.442
Leakage of urine	-.8 (1.6)	-.3 (2.0)	.220	.267
Frequent urination	-.8 (2.1)	-.3 (1.7)	.281	.244
Increased sweating	−1.3 (2.1)	-.9 (2.2)	.339	.246
Loss of sexual desire	−1.4 (2.6)	-.9 (2.6)	.393	.215
Lightheadedness	.3 (1.8)	-.2 (1.5)	.517	.164
Increased thirst	-.9 (1.9)	-.7 (1.8)	.569	.136
Increased irritability	-.4 (2.1)	-.7 (2.1)	.595	.140
Increased appetite	-.2 (2.2)	-.3 (2.0)	.849	.044
Sensitivity to heat	−1.5 (2.4)	−1.6 (2.2)	.987	.004

Self-reported data from the Weight-related Symptom Measure (WRSM). Scale from 0 (bothers not at all) to 6 (bothers a very great deal). Data are given as given as mean difference in scores (SD). P and ES-values for between-group differences.

#### The effect of weight reduction

The linear regression analyses revealed significant associations between weight reduction in per cent of baseline weight, when controlling for the effect of gender, age at baseline, age at the onset of obesity, BMI at baseline, and physical activity at baseline. The analyses of weight loss on HRQL change showed a standardized beta-coefficient of –.430 (p<.001) on the physical dimension, –.288 (p=.004) on the mental dimension, –.432 (p<.001) on the emotional dimension, .287 (p=.008) on number of symptoms, and .274 (p=.009) on reduction of symptom pressure.

## Discussion

### Key results

In this non-randomized clinical trial comparing RYGB to ILI, we found that RYGB was more effective at improving all HRQL-dimension scores (Table 2). In particular, the RYGB group had a clinically relevant effect on changes in the emotional dimension (ES=1.06) and in the physical dimension (ES=.83). Within the RYGB group all HRQL dimensions showed large improvements (ES>.80). Within the ILI group, changes were moderate (ES>.50 and <.79).

Previous studies have shown that patients treated with RYGB experience larger improvements of HRQL compared to those undergoing conventional weight loss treatment [7,8]. However, these studies did not compare the surgical procedures with a part residential lifestyle intervention program. In addition, the authors did not assess the effect sizes of the treatments on the various dimensions of HRQL.

The improvement of the emotional dimension of HRQL was particularly pronounced in the RYGB group. A possible explanation may be that the massive weight loss following RYGB after 1 year reduced the patients feeling of being fat and, accordingly, improved their feeling of being “normal”. The surgical procedure per se seems to help many patients gain control over their food intake, thus confirming the clinical observation of more “relaxed” patients one year after surgery. In addition, as suggested by Fabricatore and Wadden [29], the negative stigma associated with obesity may be caused by an undesirable body appearance and by the “character defects” other people associate with this appearance. In our terms, as patients start to experience massive weight loss, their perception of their own body is expected to improve, as is the perceptions of other people. This internal and external reduction of stigma may be followed by an improvement in self-esteem and positive emotions among obese patients experiencing massive weight loss. However, a massive weight loss and a less stigmatizable body appearance may not be the only explanations as to the improvements in the emotional HRQL. The ILI group also reported significant improvements in the emotional dimension of HRQL after 1 year, even though the effect size was moderate. The moderate effect in the ILI group may be explained by the more moderate weight loss in this group. However, weight loss may not be the only explanation. It is conceivable that the intervention itself added to the improvement of emotional HRQL in the ILI group. The group-based focus and motivational approach in the lifestyle program aimed at increasing self-efficacy, self-esteem and mood state. Previous studies seem to support this notion. Programs focusing on motivationally-oriented group sessions report as little as 3 kg. weight loss (e.g. from 103 to 100 kg.) but have found significant improvements in mood state as measured with validated psychometric instruments [30]. In another study of 440 obese patients with coronary artery disease, group support was reported to be associated with a significant improvement in the mental dimension of HRQL despite moderate weight loss [31].

The self-reported symptom scores before treatment in both groups corroborate the well-known association between high BMI, several comorbidities and physical HRQL. After 1 year we found that patients in both groups reported significantly fewer symptoms. The improvements in joint pain and physical stamina in the RYGB group were notable and may, together with improvements in skin irritation, water retention, foot problems, and shortness of breath, have resulted in easier performance of everyday personal hygiene, housekeeping, shopping and walking. All these tasks are central elements of the physical dimension of HRQL [11], which in the RYGB group showed a large effect size (ES=.83).

Another distressing obesity-associated symptom is snoring and tiredness. These symptoms were markedly reduced in the RYGB group. This finding supports a report from the SOS-study which found a substantial reduction in symptoms of sleep apnoea and daytime sleepiness in the bariatric surgery group after 2 years [32]. One might speculate that increased sleep quality and reduced daytime sleepiness may lead to increased vitality and improved functioning at work or during other daily activities, which also is embedded in the physical dimension of HRQL [11]. The finding of increased sensitivity to cold in the RYBG group is probably connected to the higher loss of fat mass with surgery [33], and is a phenomenon commonly observed within clinical practice.

The overall reduction of the number of symptoms and symptom distress in the ILI group was statistically significant, although with moderate effect sizes. However, compared to the RYGB group more patients in the ILI group were physically active at baseline, whilst the increase in physical activity after one year was larger in the ILI group than the RYGB group [9]. We believe that the combination of the overall reduction in symptom distress and higher activity levels contributed to an improvement of the physical HRQL in the ILI group, even though the weight loss was moderate. There is a consistent association of higher HRQL scores with higher levels of physical activity among healthy adults in cross-sectional studies [34], and this association is stronger on the physical dimension of the HRQL than the mental dimension [34]. We also know that interventions combining physical activity and diet improve the physical dimension of HRQL but not the mental dimension among older obese individuals with knee ostoearthritis [35].

As with the emotional and physical aspects of HRQL, the mental aspects also improved in both groups after 1 year. The RYGB group scored significantly better than the ILI group. Other studies have found similar results [7,8] between bariatric surgery and non-standardised lifestyle programs. However, our study extends previous findings to include the comparative effects of a structured, systematic part residential lifestyle program. The improvements in the mental dimension of HRQL may be explained by the greater weight loss and improvement of psychosocial status including social relations and employment opportunities [36]. A deeper understanding of the relationship between weight loss and improvement of the emotional and mental dimension of HRQL may necessitate research designs other than a quantitative approach.

We have previously shown [9] bariatric surgery to be superior to lifestyle treatment in regards to weight loss. However, the effect of weight loss on improvement of HRQL may have been moderated by the lifestyle treatment regime itself. In particular, our results suggest that a “comprehensive and multidisciplinary program intended to increase the patient’s self-efficacy in dealing with their weight problem” may impact upon HRQL, independent of weight loss.

As reported earlier our study has limitations [9]. Although preferable when conducting a clinical trial, we did not find randomization to be appropriate. According to Norwegian guidelines, treatment seeking morbidly obese subjects should be offered either conservative or surgical therapy. We therefore considered it unethical to assign patients to surgery if they qualified for a lifestyle intervention program and preferred this course of treatment to surgery. This stance also held vice versa. Thus, the differences between the groups may not be causally associated with choice of treatment. Further, the study was limited to a 1-year time span. The long term effects of the two interventions on HRQOL may differ due to intervening life events, complications of surgery, or other reasons, and these require further study.

Lifestyle intervention for morbid obesity comprises of many different methods, from very low calorie diets to comprehensive psychosocially oriented programs combining diets, physical activity and behavioral intervention. There is little robust evidence identifying the most effective lifestyle strategies for treatment and prevention of obesity in general and in morbid obesity in particular [37]. Hence, research must focus on a variety of lifestyle intervention programs in order to to identify the most beneficial treatment regimens. Our findings indicate that a pre-defined part residential multidisciplinary non-surgical weight loss program with a psychosocially-oriented motivational approach is a promising intervention when aiming to increase HRQL in morbidly obese patients. However, larger weight losses may be necessary to maximize the beneficial effects.

## Conclusion

Our study shows that following a part residential multidisciplinary lifestyle intervention program, morbidly obese patients improved their HRQL, although patients undergoing bariatric surgery experienced larger improvements in HRQL after 1 year. The higher clinical relevance of bariatric surgery on HRQL may be explained by a higher weight loss.

## Competing interests

Tor-Ivar Karlsen is a PhD-fellow at the Morbid Obesity Centre and works at the University of Agder. He is supported financially through an unrestricted educational grant from Evjeklinikken AS. All the other authors declare that there is no conflict of interest that could be perceived as prejudicing the impartiality of the research reported.

## Authors’ contributions

TIK participated in the design of the study, collected data from patients, analyzed the data and drafted the manuscript. RSL, ST, GKN revised and helped draft the manuscript, JR revised and helped draft the manuscript and assessed the statistical analyses, RS and JH designed the study and revised and helped draft the manuscript. All authors read and approved the final manuscript.
